# Prenatal diagnosis of Bardet Biedl Syndrome: A case report

**DOI:** 10.1016/j.radcr.2022.10.040

**Published:** 2022-11-14

**Authors:** Ena Arora, Aleksandr Fuks, Jessica Meyer, Judith Chervenak

**Affiliations:** a Department of Obstetrics and Gynecology, Icahn School of Medicine at Mount Sinai (NYC Health + Hospitals/Queens), 75-25,153rd street, kew garden hills, New York, NY 11367, USA; b Department of Obstetrics and Gynecology, New York University Langone Health, New York, NY, USA

**Keywords:** Bardet Biedl, Ciliopathy, Autosomal recessive, Obstructive uropathy

## Abstract

The Bardet-Biedl Syndrome (BBS), also called Laurence-Moon-Bardet-Biedl syndrome is a rare ciliopathic autosomal recessive genetic defect. BBS phenotype develops over the years and diagnosis is usually made in late childhood or early adulthood. Prenatal diagnosis is rare in absence of family history or consanguinity. The major features of this syndrome are cone-rod dystrophy, obesity, polydactyly, learning disabilities, hypogonadism in males, renal anomalies, nystagmus, speech disorders, developmental delay and ataxia. At least 20 BBS genes have been identified and all are involved in primary cilia functioning. Genetic diagnosis includes multigene sequencing technologies. Clinical management includes symptomatic treatment. In our case report, we present a case of a baby born to parents of Bengali Asian ancestry with high clinical suspicion of BBS based on fetal magnetic resonance imaging findings done during antepartum surveillance.

## Introduction

Ciliopathies are diseases caused by the dysfunction of motile and non-motile primary cilium [1]. Primary cilia are involved in numerous cellular processes, such as cell cycle control, development, migration, polarity, differentiation, stimuli transduction, proliferation, and maintenance of stem cells [[2], [3], [4]]. Ciliopathies are characterized by a high clinical and molecular heterogeneity and a large clinical overlap between entities [5]. The clinical expression of the cilia dysfunction is correlated with the activity of cilia. Motile cilia dysfunction causes hydrocephalus, infertility, chronic respiratory issues, but also congenital heart defects and organ laterality defects [6]. Non-motile cilia dysfunction determines retinal dystrophy, anosmia, hearing loss, central obesity, skeletal abnormalities (polydactyly, rib cage), hypogonadism, genital abnormalities, ataxia, epilepsy, mental disability, brain malformations, facial abnormalities, renal abnormalities, and liver disease [6]. Bardet-Biedl Syndrome (BBS) is a rare autosomal recessive multisystem non-motile ciliopathy primarily characterized by heterogeneous clinical manifestations. The prevalence of BBS is high in consanguineous populations. In the general population, BBS has a prevalence of 0.7/100,000 and prevalence at birth of 0.5/100,000 [7]. There are several reports outlining the use of ultrasound and fetal MRI in the diagnosis of BBS in the antenatal period. They are primarily retrospective reviews or involve an at-risk fetus with an affected sibling. However, echogenic kidneys observed during level II ultrasound should raise suspicion of possible BBS. The differential diagnosis of fetal echogenic kidneys is broad and is reliant on whether or not associated anomalies are visualized. Within the associated genetic syndromes, there is considerable overlap amongst the presenting features. A thorough family history should be obtained and may be useful.

### Case presentation

36 yo G4P2012 at 11w3d Asian woman of Bengali descent presented to our high risk clinic for antepartum registration. Her partner was also of Bengali descent and both denied history of consanguinity. Her obstetric history was significant for 1 spontaneous miscarriage and 2 cesarean deliveries done in 2014 for category 2 tracing and 2017 for failed trial of labor after cesarean section. Her medical history was significant for hypothyroidism and she was on Synthroid 50 µg once daily for 5 times a week and 100 µg 2 times a week. She was also diagnosed with gestational diabetes and was started on Metformin 500 mg nightly.

Her non-invasive prenatal testing was negative and consistent with female gender. Patient was lost to follow up for few months as she went to her home country.

Her anatomy scan done at 27w5d revealed fetal weight of 1062 g (25%), normal cardiac anatomy however heart appeared enlarged, fetal ascites, bladder with posterior diverticulosis and normal kidneys. Fetal ECHO done on the same day was suggestive of fetal hydrops, pericardial effusion 2 mm around anterior RV surface, no pleural effusion, fetal ascites and mild cardiomegaly.

She was admitted at 27w5d after diagnosis of fetal hydrops on anatomy scan and fetal ECHO for further management. She received 2 doses of betamethasone for fetal lung maturity. She had blood work done including serology for Parvovirus IgM and CMV IgM which was negative.

**Fig. 1:**
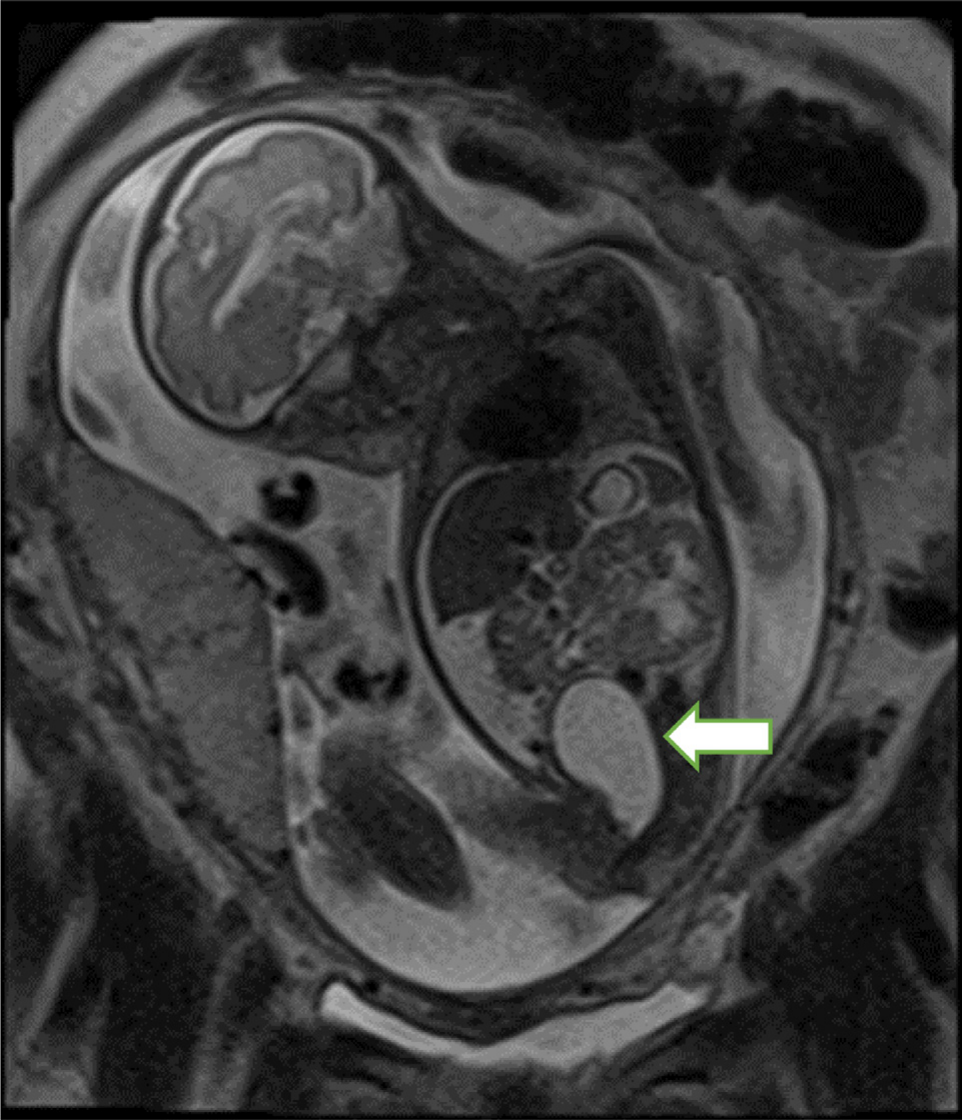
Fetal MRI at 27w6d showing ovoid pelvic cystic structure representing dilated fluid filled vagina (white arrow).

**Fig. 2:**
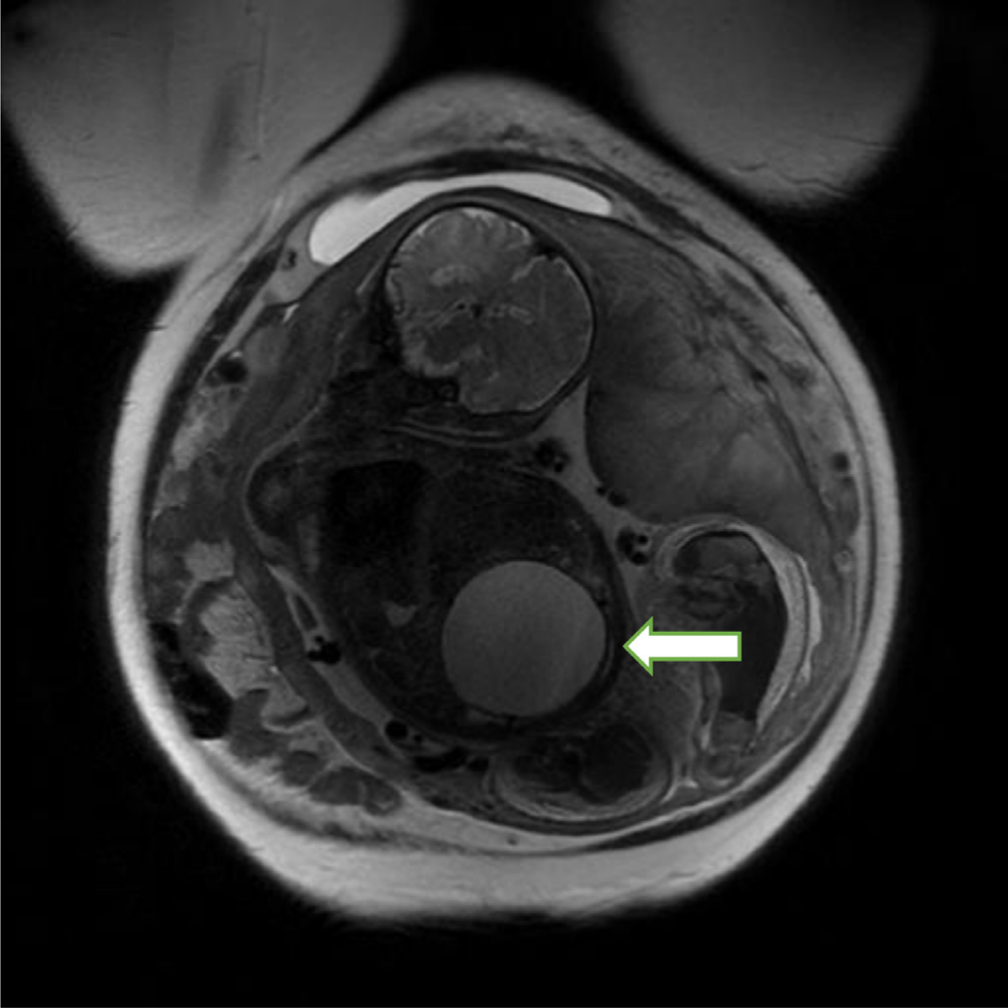
Fetal MRI at 34 weeks showing worsening obstructive uropathy (white arrow).

She underwent fetal magnetic resonance imaging (MRI) at 27w6d which noted to have midline pelvic cystic structure (posterior to urinary bladder and anterior to rectum) associated with the left urinary tract dilatation (UTD). Left renal pelvis A-P (Anterior-posterior) diameter was 7 mm, central calyceal dilatation, proximal ureteral dilatation. Mild – moderate ascites, a normal appearing rectum and possible ambiguous genitalia. Suspicion of urogenital sinus was made with impression that pelvic cystic structure represents a dilated fluid filled vagina. Normal appearing rectum ruled out the diagnosis of persistent cloaca with rectal fistula (Figs. 1 and 2).

Follow up fetal ultrasound noted to have mild to moderate ascites with no evidence of ventriculomegaly and normal middle cerebral artery dopplers, no evidence of pericardial or pleural effusion. Similar dilated cystic structure posterior to urinary bladder was seen. Left ureter was mildly dilated and left renal pelvis appeared more dilated with calyceal dilatation with A-P diameter of 10 mm consistent with UTD A 2-3. The right renal pelvis measured 4 mm which was within normal limits for the gestational age. Polyhydramnios with AFI 27 was noted. Genitalia appeared to be normal female with no evidence of bilateral club feet.

Maternal fetal medicine specialist and genetic counselor were involved in her care. Amniocentesis was offered but declined by the patient. She however agreed to do expand carrier genetic testing. Mother was diagnosed as carrier of BBS 12 and father was diagnosed with variant of uncertain significance in BBS 12. Pediatric urology consultation was done as well and possibility of urogenital sinus, possible need for fetal surgery and related prognosis were discussed. Patient desired expectant management during pregnancy and further evaluation in the postnatal period.

Serial antepartum fetal surveillance was done weekly. Serial sonograms done for Biophysical profile and fetal growth were normal.

Repeated fetal MRI done at 34 weeks noted to have anhydramnios with worsening obstructive uropathy. Findings of MRI were pelvic cystic structure measuring 6.2 × 7.1 × 6.7 cm, enlarged as compared to the prior MRI measuring same structure as 2.5 × 2.5 × 4 cm and non-visualized bladder. Left UTD with A-P diameter more than 7 mm (UTDA 2-3), peripheral calyceal dilatation in the inferior moiety, cortical thinning and ureteral dilatation, right UTD consisted of A-P diameter of more than 7 mm, ureteral dilation and peripheral calyceal dilatation, lung volume observed/expected 11% .

Maternal fetal medicine consultation was done and possibility of worsening anhydramnios leading to pulmonary hypoplasia was discussed. Patient desired expectant management and underwent tertiary cesarean section at 37 weeks. Newborn had APGARs 1/3/6/6 at 1/5/10/15 minutes, BW 2720 g and was transferred to neonatal intensive care unit on positive pressure ventilation via endotracheal tube. Ampicillin and Ceftazidime were started. Abdomen US was done and it revealed 11 cm cystic structure (dilated vagina), bladder poorly visualized, bilateral (B/L) moderate hydronephrosis with central/peripheral caliectasis. On day 2 of life, baby developed transient tachpnoea of newborn with severe pulmonary hypertension and right ventricle failure and was transitioned to extracorporeal membrane oxygenation (ECMO). On day 3 of life antibiotics were switched to vancomycin, meropenem and fluconazole due to acute drop in white blood cell count and platelets. On Day 5 and 6 of life, baby's hemodynamic status worsened with acute anuria, anasarca and electrolyte derangement, increasing abdominal distension. Abdomen US revealed decompressed bladder/vagina and loculated ascites in B/L upper quadrants. Baby was transitioned to continuous renal replacement therapy and antibiotics coverage broadened to linezolid, meropenem and fluconazole. On day 7 and 8, baby had scant urine output with worsening abdominal distension and maintained on vasopressors. On day 9 of life, baby had continued hemolysis secondary to ECMO. Goals of care discussion were held and care was withdrawn after parental decision, leading to neonatal death on day 9.

Unfortunately, no genetic testing or autopsy could be done on the baby as the parents did not consent. But based on the parental carrier testing done by the prenatal genetic counsellor during the pregnancy, the mother was found to be a carrier of BBS 12. The father was not reported as a carrier of BBS 12. The father however was found to have a variant of uncertain significance in BBS 12. Taking into account our clinical impression and the possible carrier status in BBS 12 of both the parents, it is likely that baby had BBS 12.

## Discussion

In 1866, Laurence and Moon described a family of 4 siblings with retinal dystrophy, obesity, spastic paraparesis and cognitive defect [8]. Bardet [9] and Biedl [10] later reported separately on further similarly affected individuals who in addition had post-axial polydactyly and the condition was coined Laurence-Moon-Bardet-Biedl syndrome. It is a rare ciliopathic, pleiotropic autosomal recessive defect, mostly occurs in children born from consanguineous marriages.

The major features of this syndrome are cone-rod dystrophy, polydactyly, obesity, learning disabilities, hypogonadism in males, renal anomalies, nystagmus, speech disorders, developmental delay, polyuria/polydipsia, ataxia, and poor coordination/clumsiness. Incidence rates in North America and Europe vary from 1:140,000 to 1:160,000 live births. Conversely, in Kuwait and Newfoundland, the rate is much greater, with an estimated frequency of 1:13,500 and 1:17,500, respectively [11].

To date, pathogenic variants in 26 genes have been shown to be involved in the molecular basis of this rare ciliopathy. Of these causal loci, *BBS12* accounts for 8% of all cases. In this type of inheritance, both parents will be carriers, meaning they have one gene for the syndrome paired with one normal gene. Each of their children then has a 25% chance of inheriting the 2 Bardet–Biedl genes (one from each parent) needed to cause the disorder. Carriers are unaffected because they have only one copy of the gene.

With earlier exposure to prenatal care along with the increased accessibility and use of ultrasound/fetal MRI in the antenatal period, more anomalies are being identified earlier in pregnancy. In patients that are at high risk for certain disorders, these anomalies can provide sufficient evidence that a particular disease is present without the need for further testing.

Dar et al. describe the diagnosis of BBS via a targeted ultrasound at 16 weeks revealing postaxial polydactyly in a pregnancy of a woman with 2 prior children affected with the disorder [12]. With the presumed diagnosis the patient decided to terminate the pregnancy.

Cassart et al. looked retrospectively at 127 patients with hyperechoic kidneys, targeting 11 who ultimately were diagnosed with BBS via clinical manifestations [13]. They confirmed that in a family with known history of BBS, ultrasound findings of polycystic kidneys with or without polydactyly confer a very high likelihood of recurrence. Through their review they noted that BBS should be considered as a differential diagnosis in those with echogenic kidneys without a known family history. This information should be included in further evaluations as well as genetic counseling to help guide decision about invasive testing and what method of genetic analysis should be pursued.

Karmous-Benailly et al. evaluated 13 patients with polycystic kidneys and polydactyly by prenatal ultrasound for evidence of mutations related to BBS [14]. Six patients were found to have homozygous mutations, 3 with compound heterozygous mutations, and 4 without any mutations identified. The shortcoming of this study was that only known BBS mutations were tested for, and it is expected that at least 20% of patients with BBS will not have one of these mutations.

Beales et al. have given a diagnostic criterion for BBS: the presence of either 4 primary features or a combination of 3 primary and 2 secondary features [15]. The presence of 4 primary features on their own or 3 primary coupled with 2 secondary features are the clinical grounds for making a diagnosis. Cone-rod dystrophy, polydactyly, obesity, learning disabilities, hypogonadism in males, and renal anomalies are classified as primary features, whereas secondary features include speech disorders, brachydactyly, developmental delay, polyuria/polydipsia, ataxia, poor coordination/clumsiness, diabetes mellitus, left ventricular hypertrophy, hepatic fibrosis, spasticity, and hearing loss. Apart from these features, short stature, crowding of teeth, hypermobile or lax joints, and early osteoarthritis are also reported [16]. Confusion still exists laurence moon syndrome (LMS) and BBS. Pigmentary retinal degeneration, mental retardation, and hypogonadism are common in both, whereas spastic paraplegia is predominant in LMS and polydactyly and obesity are seen in BBS. Because of some common features, some researchers believe BBS to be a part of LMS.

BBS is a chronic disease without any specific cure. Parents require proper counselling with proper advice regarding the life-threatening systemic complications of the syndrome. The treatment for BBS is mainly symptomatic. Physical therapy and exercise can reduce the symptoms of spasticity. A dedicated regimen of nutritious, well-balanced meals and regular exercise is recommended, as there is an increased incidence of diabetes and abnormal cholesterol levels. A low protein diet also slows the progression of renal diseases in BBS [17]. The poor functional capacity of the anterior pituitary gland, resulting in slow metabolism, poor growth, and impaired fertility, can be managed with hormone replacement therapies. Levothyroxine can aid in increasing the body metabolism, resulting in reduced lethargy, hair loss, and obesity. Growth hormone supplementation reduces the psychosocial burden of short stature, whereas testosterone supplementation can be given in patients with markedly low levels to prevent underdeveloped genitalia. Accessory digits are generally nonfunctional and can be removed for cosmetic purposes. Typically, retinal dystrophy is the first symptom that arises before the age of 10 years but affects almost all patients below the age of 20 years [18]. Glasses can be used to treat this, and regular ophthalmologist visits are recommended [19]. A course of Vitamin A may be given for nyctalopia. Speech therapy and proper rehabilitation may be necessary for some children affected by this syndrome. A pediatrician should be involved in the overall care of the patient and should refer the patient to appropriate subspecialities when necessary.

## Conclusion

BBS is a rare syndrome requiring early diagnosis and expert multidisciplinary management as it is associated with life threatening complications. Unique presentation in our case was rare prenatal suspicion and diagnosis of BBS with help of antepartum ultrasound and fetal MRI imaging. Prenatal diagnosis of BBS can be done with ultrasound, fetal MRI and genetic testing. Parents require adequate counseling and proper advice regarding life threatening systemic complications of the syndrome.

## Patient consent

This is to verify that appropriate informed consent was obtained from the patient for this report.

## References

[1] Strong A., Li D., Mentch F., Bedoukian E., Hartung E.A., Meyers K. (2021). Ciliopathies: coloring outside of the lines. Am. J. Med. Genet. Part A..

[2] Cardenas-Rodriguez M., Badano J.L. (2009). Ciliary biology: understanding the cellular and genetic basis of human ciliopathies. Am. J. Med. Genet. Part C Semin. Med. Genet..

[3] Christensen S.T., Morthorst S., Mogensen J.B., Pedersen L.B. (2017). Primary cilia and coordination of receptor tyrosine kinase (RTK) and transforming growth factor β (TGF-β) signaling. Cold Spring Harb. Perspect. Biol..

[4] Álvarez-Satta M., Cástro-Sánchez S., Valverde D. (2017). Bardet-Biedl syndrome as a chaperonopathy: dissecting the major role of chaperonin-like BBS proteins (BBS6-BBS10-BBS12) *Front*. Mol. Biosci..

[5] Zaki M.S., Sattar S., Massoudi R.A., Gleeson J.G. (2011). Co-occurrence of distinct ciliopathy diseases in single families suggests genetic modifiers. Am. J. Med. Genet. Part A..

[6] Reiter J.F., Leroux M.R. (2017). Genes and molecular pathways underpinning ciliopathies. Nat. Rev. Mol. Cell Biol..

[7] Florea L, Caba L, Gorduza EV (2021). Bardet–Biedl Syndrome—Multiple Kaleidoscope Images: Insight into Mechanisms of Genotype—Phenotype Correlations. Genes.

[8] Laurence JZ, Moon RC. (1995). Four cases of ‘retinitis pigmentosa' occurring in the same family, and accompanied by general imperfections of development. Obes Res.

[9] Bardet G. (1995). On congenital obesity syndrome with polydactyly and retinitis pigmentosa (a contribution to the study of clinical forms of hypophyseal obesity. Obes Res.

[10] Biedl A. (1995). A pair of siblings with adiposo-genital dystrophy. Obes Res.

[11] Katsanis N, Lupski JR, Beales PL. (2001). Exploring the molecular basis of Bardet-Biedl syndrome. Hum Mol Genet.

[12] Dar P., Sachs G.S., Carter S.M., Ferreira J.C., Nitowsky H.M, Gross S.J (2001). Prenatal diagnosis of Bardet-Biedl syndrome by targeted second-trimester sonography. Ultrasound Obstet Gynecol.

[13] Cassart M., Eurin D., Didier F., Guibaud L., Avni E.F. (2004). Antenatal renal sonographic anomalies and postnatal follow up of renal involvement in Bardet-Biedl syndrome. Ultrasound Obstet Gynecol.

[14] Karmous-Benailly H., Martinovic J., Gubler M.C., Sirot Y., Clech L., Ozilou C. (2005). Antenatal presentation of Bardet-Biedl syndrome may mimic Meckel syndrome. Am J Hum Genet.

[15] Beales PL, Elcioglu N, Woolf AS, Parker D, Flinter FA. (1999). New criteria for improved diagnosis of Bardet-Biedl syndrome: results of a population survey. J Med Genet.

[16] Abbasi A, Butt N, Sultan B, Munir SM. (2009). Hypokalemic paralysis and megaloblastic anemia in Laurence-Moon-Bardet-Biedl syndrome. J Coll Physicians Surg Pak.

[17] Dervisoglu E, Isgoren S, Kasgari D, Demir H, Yilmaz A. (2011). Obesity control and low protein diet preserve or even improve renal functions in Bardet-Biedl syndrome: a report of two cases. Med Sci Monit.

[18] Fulton AB, Hansen RM, Glynn RJ. (1993). Natural course of visual functions in the Bardet-Biedl syndrome. Arch Ophthalmol.

[19] Sumir K, Bharat BM, Jyotistema M (2012). Bardet-Biedl syndrome: a rare case report from North India. Indian J Dermatol Venereol Leprol.

